# Relationship Between Psychological Factors and Health-Related Quality of Life in Patients with Chronic Low Back Pain

**DOI:** 10.3390/healthcare12242531

**Published:** 2024-12-15

**Authors:** Iva Dimitrijević, Dijana Hnatešen, Ivan Radoš, Dino Budrovac, Marija Raguž

**Affiliations:** 1 Clinical Department of Pain Management, University Hospital Osijek, 31000 Osijek, Croatia; hnatesen@yahoo.com (D.H.); irados11@gmail.com (I.R.); dino.budrovac@mefos.hr (D.B.); 2 Faculty of Medicine, Josip Juraj Strossmayer University of Osijek, 31000 Osijek, Croatia; maraguz@mefos.hr; 3 Nursing Institute “Professor Radivoje Radić”, Faculty of Dental Medicine and Health Osijek, Josip Juraj Strossmayer University of Osijek, 31000 Osijek, Croatia

**Keywords:** chronic low back pain, health-related quality of life, psychological factors

## Abstract

Background: Low back pain has frequently been mentioned as the most common sort of chronic pain, and numerous studies have confirmed its influence on the health-related quality of life (HRQoL). Despite a great deal of research demonstrating the important part that psychological factors play in explaining HRQoL, a therapeutic setting that prioritizes the physical domain still predominates. For this reason, the aim of this study is to assess the relationship between age, pain intensity, pain catastrophizing, depression, anxiety, pain-related anxiety, chronic pain acceptance and the psychological and physical dimensions of HRQoL in patients with chronic low back pain (CLBP). Methods: Data were collected from 201 patients with CLBP using sociodemographic data, the SF-36 Health Status Questionnaire (SF-36), the Hospital Anxiety and Depression Scale (HADS), the Pain Anxiety Symptoms Scale Short Form 20 (PASS-20), the Pain Catastrophizing Scale (PCS), the Chronic Pain Acceptance Questionnaire (CPAQ-8) and the Numeric Pain Rating Scale (NRS). The linear regression model for the dependent variable of Physical Health (SF-36 PhyH) was statistically significant (F (7, 201) = 38.951, *p* < 0.05), explaining 57.6% of the variance regarding the Physical Health dimension of HRQL in patients with CLBP. Results: The linear regression model for the dependent variable of Psychological Health (SF-36 PsyH) was statistically significant (F (7, 200) = 39.049, *p* < 0.05), explaining 57.7% of the variance regarding the Psychological Health dimension of HRQL in patients with CLBP. Conclusions: The findings of this study confirm that age, pain intensity, depression, pain-related anxiety and chronic pain acceptance are significant predictors of the physical dimension of HRQoL, while pain intensity, anxiety and depression proved to be significant predictors of the psychological dimension of HRQoL in patients with CLBP.

## 1. Introduction

The International Association for the Study of Pain (IASP) definition of pain, which underwent its last revision in 2020, describes pain as “an unpleasant sensory and emotional experience associated with actual or potential tissue damage; or similar to that experience associated with actual or potential tissue damage”, which includes the significant statement that pain is always an individual experience that is influenced by biological, psychological and social factors [1]. The traditional biomedical approach is ineffective in assessing the psychosocial and behavioral mechanisms that can change the manifestation and maintenance of pain [2,3]. According to Fillingim’s biopsychosocial model of pain, the painful stimulus is processed through the biopsychosocial context of the individual, which leads to the experience of pain [4].

Chronic pain (CP) is defined as pain that lasts or recurs for more than three months [5]. It represents a significant public health problem reported by about 20% of adults in the world and is one of the most common reasons people seek medical help [6]. Low back pain (LBP) is described anatomically as reaching from the iliac crest to the 12th rib [7] *and* has frequently been mentioned as the most common sort of chronic pain [8,9]. Although many episodes of LBP improve significantly within 6 weeks, and 33% of patients recover in the first 3 months, research has shown that 65% of patients continue to report pain after 12 months [10,11]. Furthermore, up to 33% of patients will have a relapse within one year after recovering from the previous episode [10,12]. According to the Global Burden of Disease Study from 2017, LBP included 577 million people, including all age groups and genders, and women had a higher prevalence than men [13]. A similar finding related to gender prevalence was established by a systematic investigation of 28 research studies involving data from 488,000 participants by Meucci et al., according to which the prevalence of LBP was 50% higher in women than in men worldwide [14]. In 2017, the Global Burden of Disease Study found that the most common cause of disability among women globally was low back pain [15,16]. Anatomical variations, pregnancy-related changes and hormonal changes during menstruation could all be contributing factors to this increased frequency in women [16]. Studies indicate that variations in hormone levels, specifically those of estrogen and progesterone, impact how pain is perceived and responded to [15,16]. Furthermore, lower back pain may result from biomechanical changes in the spine and pelvis brought about by hormonal changes during pregnancy [15]. In 2020, LBP affected 619 million people worldwide, and it is estimated that the number of cases will increase to 843 million by 2050, mainly due to population growth and aging [13]. Research shows a negative impact of CLBP on the QOL [17,18].

Quality of life (QOL) is a broad concept that includes all aspects of an individual’s life, while health-related quality of life (HRQoL) focuses on aspects of quality of life related to an individual’s health [19], including the level of a person’s daily functioning and the ability to live a fulfilled life [20]. Numerous studies have confirmed the influence of CLBP on HRQoL in a variety of life domains, including physical and mental health, social relationships and functional abilities [9,19,21]. Psychological factors are also an important predictor for evaluating the effectiveness of CLBP treatment [22]. Hong et al. studied depression, anxiety, disability and HRQoL in patients with CLBP and found that these patients had significant functional disability, significantly impaired psychological status and impaired HRQoL [23]. Also, the research by Hnatešen et al. showed the existence of impaired HRQoL and significant emotional distress in patients with CLBP [17]. Therefore, some of the psychological factors, such as anxiety [24], depression [25], pain catastrophizing [26,27] and pain acceptance [26], are recognized as important predictors of HRQoL. A systematic review by Agnus Tom et al. pointed out the psychological status of the individual as a significant contributor to the QOL of individuals with CLBP. Numerous psychological factors, such as kinesiophobia, pain and fear avoidance beliefs, self-efficacy, anxiety, coping mechanisms, sleep quality, locus of control and catastrophizing, were found to be significant QOL determinants in people with CLBP [19].

Despite the existence of numerous studies that have identified individual psychological factors as predictors of impaired HRQoL, a clinical environment that focuses more on the physical aspect still prevails. Hence, the aim of this study is to assess the relationship between pain intensity, pain catastrophizing, depression, anxiety, pain-related anxiety, pain acceptance and HRQoL in patients with CLBP with the goal of supporting existing findings and raising awareness of the importance of a biopsychosocial approach in the treatment of CLBP. Also, the research aims to emphasize the importance of psychological interventions in the treatment of CLBP.

## 2. Materials and Methods

### 2.1. Participants

**Figure 1:**
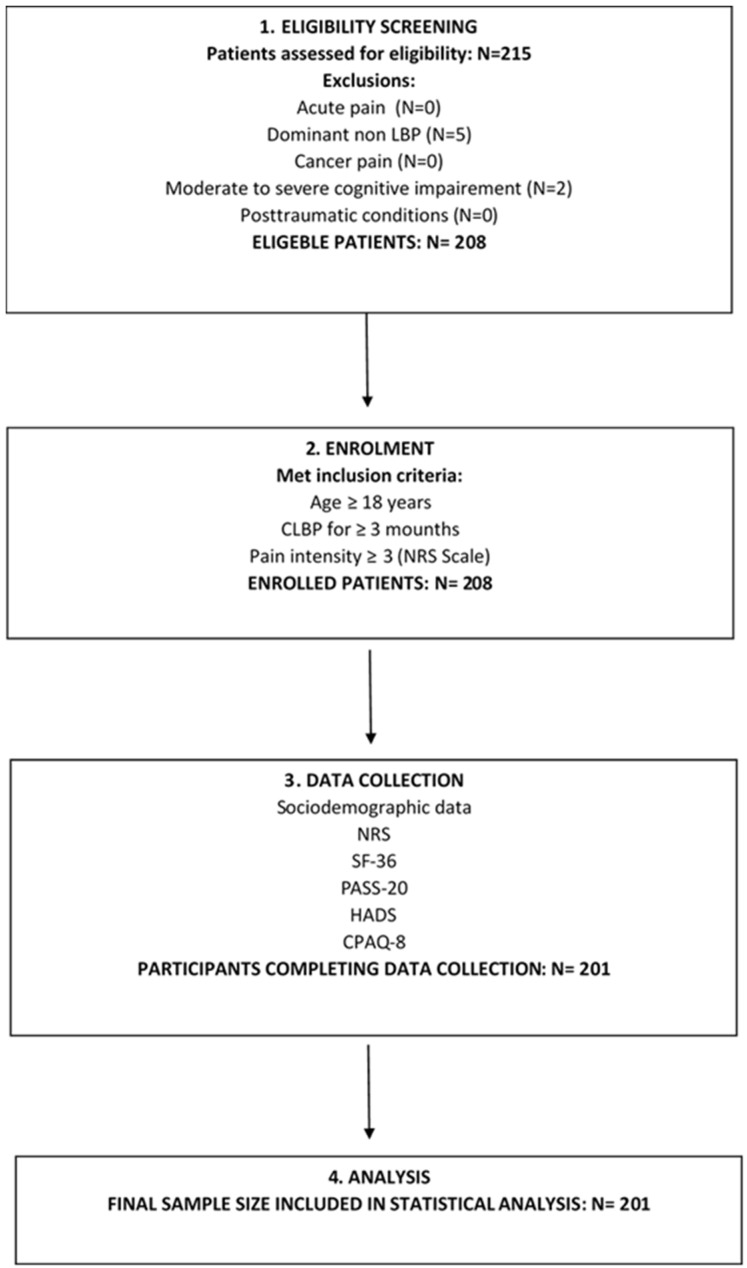
Study design.

This cross-sectional study was conducted at the Clinical Department of Pain Management at the University Hospital Osijek between March 2023 and May 2023. Using the G*Power program [28], the minimum required sample size for the purposes of conducting regression analysis was calculated. For a medium effect size with a significance level of 0.05 and a power of 0.80, the minimum sample size was 102. Data were collected from 201 patients with CLBP who came for a medical examination at the outpatient clinic for pain treatment (Figure 1). The inclusion criteria were participants 18 years of age or older with CLBP (≥3 months) with the intensity of 3 or more as assessed by the NRS scale. The diagnosis of CLBP was made by a physician during the examination and through the anamnesis and status of the patient. The exclusion criteria were acute pain, dominant pain in part of the body other than the lower back and cancer pain, the presence of a psychotic disorder and moderate-to-severe cognitive impairment and post-traumatic conditions. During the examination and analysis of the patient’s medical documentation, the physician determined the absence of psychotic processes and moderate-to-severe cognitive impairment. Participants were also asked for their written consent to voluntary participation in the study. The study was conducted with the approval of the Ethics Committee (R1-11662-2/2022.) and in accordance with the Declaration of Helsinki.

### 2.2. Measures

All participants were asked to complete the sociodemographic data, the SF-36 Health Status Questionnaire (SF-36), the Pain Anxiety Symptoms Scale Short Form 20 (PASS-20), the Hospital Anxiety and Depression Scale (HADS), the Chronic Pain Acceptance Questionnaire (CPAQ-8) and the Numeric Pain Rating Scale (NRS).

Sociodemographic data collected information about gender, age, residence, education level, working status, marital status and duration of pain.

The SF-36 Health Status Questionnaire (SF-36) was used to evaluate health-related quality of life. It consists of eight subscales: physical functioning, bodily pain, role limitations due to physical health, role limitations due to emotional problems, mental health, social functioning, vitality and general health. Health-related quality of life was assessed using the SF-36 Health Status Questionnaire (SF-36). Physical functioning, bodily pain, role limitations due to physical health, role limitations due to emotional problems, mental health, social functioning, vitality and general health are its eight subscales. The SF-36 questionnaire represents two general concepts of health: physical health (PhyH) and psychological health (PsyH) dimensions of HRQoL. The subscales evaluate health between 0 and 100, where 0 indicates poor health and 100 good health [29]. The SF-36 questionnaire demonstrated good psychometric properties on the representative sample of Croatian adult population [30]. In the current study, Cronbach’s alpha was acceptable for all subscales. The lowest Cronbach’s alpha was recorded for the general health subscale, which was 0.72, while the highest Cronbach’s alpha was recorded for the role limitations due to emotional problems subscale, which was 0.91.

The Pain Anxiety Symptoms Scale Short Form 20 (PASS-20) was used to evaluate pain-related anxiety. The PASS-20 scale assesses pain-specific anxiety symptoms and consists of four 5-item subscales measuring cognitive anxiety responses, escape and avoidance, fearful thinking and physiological anxiety responses. Pain-related anxiety was assessed using the Pain Anxiety Symptoms Scale Short Form 20 (PASS-20). Four 5-item subscales evaluating cognitive anxiety responses, escape and avoidance, fearful thinking and physiological anxiety responses are part of the PASS-20 scale. All items are rated on a frequency scale from 0 (never) to 5 (always). The total score is obtained by adding the sum of rounded values. A higher score indicates greater pain-related anxiety [31]. The PASS-20 scale demonstrated good psychometric properties on the CLBP sample, with Cronbach’s alpha values for the subscales and the total score ranging from 0.70 to 0.91 [32]. In the current study, PASS-20 also demonstrated good psychometric properties, with values ranging from 0.86 to 0.95.

The Pain Catastrophizing Scale (PCS) is a measure of catastrophizing associated with the experience of pain. It consists of 13 items, in which the respondent’s task is to recall the last painful experience and mark on a Likert scale the extent to which they felt in relation to the statements. The responses are ranked from 0 (never) to 4 (all the time). The PCS includes three subscales: rumination, exaggeration and helplessness. The total score is obtained by adding the sum of rounded values, with a range of results from 0 to 52. A higher result denotes a higher level of catastrophizing. Given that all three subscales are highly correlated, a total score is generally used [33]. The Croatian version of PCS showed the same 3-factor structure (rumination, magnification and helplessness). It also showed appropriate internal consistency, with Cronbach’s alpha of 0.88 [34]. In the current study, Cronbach’s alpha was 0.95.

The Hospital Anxiety and Depression Scale (HADS) was used to measure anxiety and depression. The scale consists of 14 items: 7 related to anxiety and 7 to depression. It is scored with 4-point (0–3) Likert-type responses, with 3 denoting the highest anxiety or depression level; therefore, the possible scores ranged from 0 to 21 for anxiety and from 0 to 21 for depression [31]. There are 3 categories of symptom intensity, with a score lower than 7 being interpreted as asymptomatic, a score of 8–10 indicating mild or moderate symptoms and a score of 10 or more suggesting clinically significant symptoms [32]. The HADS demonstrated good psychometric properties, with Cronbach’s alpha values for the subscales ranging from 0.68 to 0.93 for anxiety and from 0.67 to 0.90 for depression [33]. In the current study, Cronbach’s alpha was 0.88 for anxiety and 0.83 for depression.

The Chronic Pain Acceptance Questionnaire (CPAQ-8) was used to measure chronic pain acceptance. The CPAQ-8 questionnaire consists of two subscales, each assessing a different aspect of pain acceptance. Activity engagement (AE) assesses the degree to which respondents report being active with the continuing experience of pain. Pain willingness (PW) assesses the degree to which respondents report being open to the experience of pain without the need to engage in unsuccessful pain control efforts. It is scored on a Likert scale (0–6), with lower scores indicating lower levels of AE and PW [34]. All CPAQ-8 scales demonstrated good internal consistency, with Cronbach’s alpha higher than 0.80 [35]. In the current study, Cronbach’s alpha was 0.74 for PW and 0.83 for AE.

The Numeric Pain Rating Scale (NRS) was used to measure the intensity of pain. The scale consists of a solid line bounded at the beginning and end of its length by numbers from 0 to 10. On the far left is number 0, which indicates the absence of pain, while on the far right is number 10, which indicates unbearable pain [36].

Anxiety and depression were measured using the Hospital Anxiety and Depression Scale (HADS). There are 14 items on the scale: 7 relating to anxiety and 7 to depression. The possible scores are from 0 to 21 for anxiety and for depression, since they are scored using 4-point (0–3) Likert-type replies, where 3 represents the maximum level of either anxiety or depression [35]. A score of less than 7 is considered asymptomatic; a score between 8 and 10 indicates mild or moderate symptoms; and a score of 10 or more indicates clinically severe symptoms [36]. With Cronbach’s alpha values for the anxiety and depression subscales ranging from 0.68 to 0.93 and from 0.67 to 0.90, respectively, the HADS showed good psychometric characteristics [37]. Cronbach’s alpha for anxiety and depression in the current study was 0.88 and 0.83, respectively.

Acceptance of chronic pain was measured using the Chronic Pain Acceptance Questionnaire (CPAQ-8). Each of the two subscales that comprise the CPAQ-8 questionnaire measures specific aspects of pain acceptance. The measure of activity engagement (AE) evaluates how active respondents say they are despite their ongoing pain. The measure of pain willingness (PW) evaluates how much respondents say they are willing to tolerate pain without the use of ineffective pain management techniques. A Likert scale (0–6) is used for scoring, where lower scores correspond to lower degrees of AE and PW [38]. With Cronbach’s alpha greater than 0.80, all CPAQ-8 scales showed strong internal consistency [39]. Cronbach’s alpha for PW and AE in the current study was 0.74 and 0.83, respectively.

The severity of pain was assessed using the Numeric Pain Rating Scale (NRS). The scale is composed of a solid line, with numbers ranging from 0 to 10 at either end. Number 0 denotes a lack of pain on the far left, and number 10 denotes excruciating pain on the far right [40].

### 2.3. Statistical Analysis

Statistical analysis was performed using the IBM SPSS Statistics (release 24.0.0.0; IBM Corp., 2016; IBM SPSS Statistics for Windows, IBM Corp., Armonk, NY, USA) software tools, with statistical significance defined as *p* < 0.05. IBM SPSS Statistics (version 24.0.0.0; IBM Corp., 2016; IBM SPSS Statistics for Windows, IBM Corp., Armonk, NY, USA) software tools were used for statistical analysis, and *p* < 0.05 was considered statistically significant. All data were tested for normality with skewness and kurtosis. After normality analysis, descriptive statistics were calculated for patient sociodemographic characteristics and for all the applied scales (SF-36, PASS-20, HADS, CPAQ-8 and PSC). Pearson’s correlation coefficient and linear regression analysis were conducted to examine the relationship between SF-36 (PhyH and PsyH) and the other variables (age, NRS, PASS-20, HADS, PCS and CPAQ-8).

## 3. Results

This study involved 201 patients who met the inclusion criteria, agreed to participate, signed the informed consent form and answered the questionnaires. Sociodemographic characteristics, such as gender, age, education level, working status, marital status and duration of pain, are shown in Table 1. The mean age was 54.36 +/− 12.072 years (in a range from 27 to 82 years). The majority of the sample were female (78.6%), living in urban areas (65.7%), employed (63.1%), married (68.1%), with secondary education (67.7%) and with CLBP for more than 7 years (51.3%).

The data were tested for normality with skewness and kurtosis. Their result proved to be satisfactory for conducting parametric statistics. A summary of the scores of the applied questionnaires is presented in Table 2.

Table 3 outlines Pearson’s correlation coefficients among the measured variables. Significant correlations were found between the SF-PhyH and SF-PsyH dimensions of HRQoL. Moreover, significant correlations were also found between SF-PhyH and age, NRS, PSC, PASS-20, CPAQ-8, HADS-A and HADS-D. Significant correlations were also found between SF-PsyH and NRS, PSC, PASS-20, CPAQ-8, HADS-A and HADS-D.

**Figure 2:**
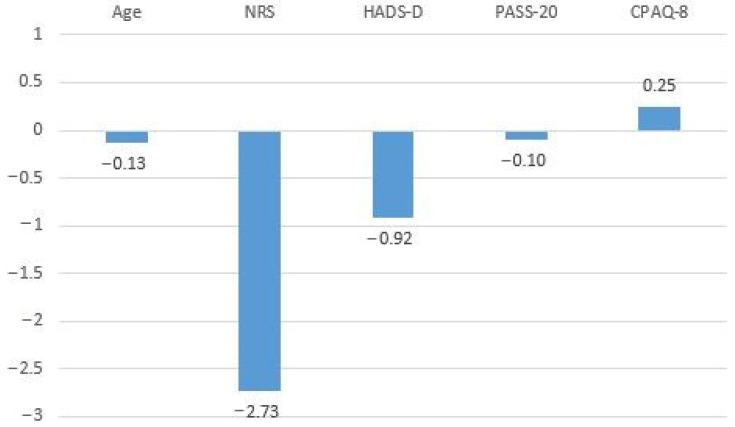
B coefficients of linear regression analysis for the dependent variable SF-PhyH.

Furthermore, linear regression analysis with a 95% confidence interval was conducted to examine the relationship between SF-PhyH and SF-PsyH as the dependent variables and all the other variables (age, NRS, PASS-20, HADS-A, HADS-D, PCS and CPAQ-8). The regression coefficients of the predictors for the dependent variable SF-PhyH are shown in Table 4. A significant model emerged (F (7, 201) = 38.951, *p* < 0.05), explaining 57.6% of the variance in SF-PhyH. Age, NRS, HADS-D, PASS-20 and CPAQ-8 contributed significantly to this model. The B coefficients of linear regression analysis for the dependent variable SF-PhyH are shown in Figure 2.

**Figure 3:**
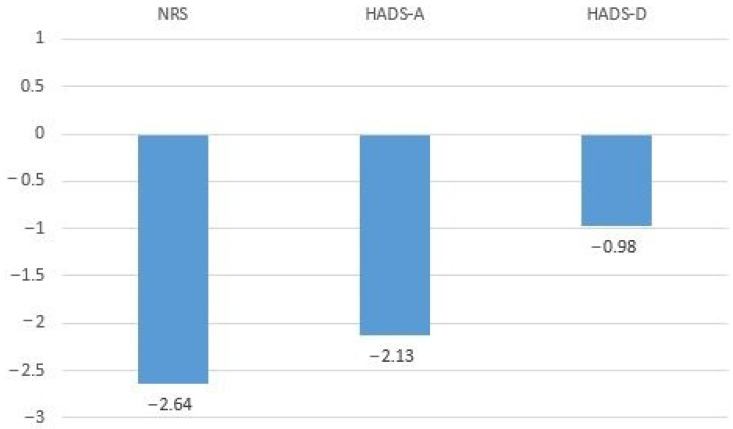
B coefficients of linear regression analysis for the dependent variable SF-PsyH.

The regression coefficients of the predictors for the dependent variable SF-PsyH are shown in Table 5. A significant model also emerged (F (7, 200) = 39.049, *p* < 0.05), explaining 57.7% of the variance in SF-PhyH. The following variables significantly contributed to this model: NRS, HADS-A and HADS-D. The B coefficients of linear regression analysis for the dependent variable SF-PsyH are shown in Figure 3.

## 4. Discussion

The aim of this study was to investigate whether pain intensity, pain catastrophizing, depression, anxiety, fear of pain and pain acceptance can predict the physical and psychological dimensions of HRQoL in patients with CLBP. The study’s results showed that age, pain intensity, depression, pain-related anxiety and chronic pain acceptance are significant predictors of the physical dimension of HRQoL, while pain intensity, anxiety and depression proved to be significant predictors of the psychological dimension of HRQoL in patients with CLBP.

This study’s sample consisted mainly of female participants. This ratio by gender can be expected because studies show a higher prevalence of CLBP in women [14,41]. The majority of participants had completed their secondary education, which matched the proportion of the adult population of the Republic of Croatia [42].

The findings of this study identified the existence of moderate intensity of pain and a lower self-assessment of the physical than the psychological dimension of HRQoL in patients with CLBP. These findings are in agreement with other studies [43,44]. For example, Stefane et al. also found moderate pain intensity and greater impairment in the physical dimension of QOL in CLBP patients [44].

Furthermore, the results of the study indicate a statistically significant correlation between the psychological and physical dimensions of HRQoL and all the investigated psychological factors: pain catastrophizing, pain-related anxiety, chronic pain acceptance, depression and anxiety. Numerous studies have already confirmed such findings and highlighted the connection between the mentioned psychological factors and the HRQoL of patients with CLBP [9,19,21,25,26,27]. As expected, this study found a positive correlation between pain acceptance and HRQoL and a negative correlation between pain catastrophizing and HRQoL, which is in accordance with the research findings from the study conducted by authors Semeru and Halim. Their explanation of such results is that acceptance provides the patient with physical pain relief, which improves their QOL. On the other hand, people who catastrophize about their pain have a lower QOL and are more likely to become angry, upset and worried about their pain [26].

In the current study, pain intensity was proved to be a strong predictor of the physical and psychological dimensions of HRQoL in patients with CLBP. This finding agrees with the research by Mutubiki et al., who found a significant negative longitudinal relationship between NRS and HRQoL [45]. A similar result was obtained by Wettstein et al., where older patients, despite significant physical disability, achieved better results in assessments of psychological health and well-being compared to younger patients [46]. These findings can be explained by the “well-being paradox”, according to which there does not have to be a connection between cognitive and physical declines and disability and subjective well-being in older adults [47].

Looking separately at the psychological and physical dimensions of HRQoL, an interesting result was obtained in the research. It was shown that more psychological factors were significant predictors of the physical dimension of HRQoL, while fewer psychological factors significantly predicted the psychological dimension of HRQoL. Thus, in the prediction of the physical dimension of HRQoL, pain intensity stood out as the most important predictor, followed by depression, pain-related anxiety, chronic pain acceptance and age as a demographic factor. In the explanation of the variance in the regression model of the psychological dimension of HRQoL, anxiety proved to be the strongest predictor. Along with it, the intensity of pain and depression also proved to be significant. Surprisingly, age, pain-related anxiety and chronic pain acceptance were not significant predictors of the psychological dimension of HRQoL. Regarding age and HRQoL, in this study, age was shown to be a significant predictor of only the physical dimension of HRQoL. Other studies also give inconsistent results. Some studies have identified age as a significant predictor of the psychological health dimension of HRQoL [46,48], while another study revealed that age does not affect any dimension of QOL [49]. Although research finds a connection between depression and anxiety and HRQoL [50,51,52], in this study, only depression was found to be a significant predictor of the physical dimension of HRQoL. Nevertheless, both anxiety and depression were found to be significant predictors of the psychological dimension of HRQoL. This finding can be explained by the significant influence of physiological symptoms of depression, such as fatigue and a lack of energy, on the physical dimension of HRQoL. Similar to the obtained result, in the research conducted by Hung et al., depression was the most powerful factor associated with disability in CLBP subjects among depression, anxiety and somatic symptoms [53]. As mentioned earlier, pain-related anxiety proved to be a significant predictor of only the physical dimension of HRQoL. A meta-analytic review from Burke et al. confirms a significant level of pain-related anxiety in patients with CP compared to healthy individuals [54]. However, no studies investigating pain-related anxiety as a predictor of HRQoL were found. The result of the study can be explained by the significant influence of pain-related fear on physical activity and, consequently, on the physical dimension of the HRQoL of patients with CLBP. A similar explanation is offered by Marshall et al., who emphasize the importance of fear and depression as factors that influence the physical disability of CLBP patients [55]. Furthermore, the same trend as with pain-related anxiety was also found with the chronic pain acceptance factor, which proved to be a significant predictor of only the physical dimension of HRQoL. In contrast, the research by Viane et al. showed that acceptance of pain predicted mental well-being but did not account for physical functioning [56]. It can be assumed that in this study, chronic pain acceptance played an important role in participation in activities despite the pain and led to a better assessment of the physical dimension of HRQoL. This explanation is similar to the perspective of Semeru et al., whose study investigated the relationship between chronic pain acceptance and the overall quality of life of patients with CLBP and found that acceptance increased the patients’ quality of life by giving physical relief from pain [26]. Unlike some research works that emphasized the importance of pain catastrophizing as a predictor of QOL in patients with CLBP [26,57], in this research, pain catastrophizing was not found to be a significant predictor of any HRQoL dimension. Although the research found a statistically significant correlation between pain catastrophizing and both dimensions of HRQoL, it did not stand out as a significant predictor in the linear regression analysis. It can be assumed that pain catastrophizing had a moderating effect on the other factors included in the model, such as anxiety, depression, pain-related anxiety and chronic pain acceptance. In accordance with this assumption, some studies using regression analysis found that catastrophizing and depression are significantly associated [58,59].

There are some limitations to this study that should be emphasized, such as the cross-sectional design and the sociodemographic characteristics of the sample, such as the uneven distribution of participants by gender. Moreover, the study did not investigate the relationship between the duration of CLBP, the use of medications, the presence of other chronic diseases and HRQoL. Also, some psychological factors, such as personality traits, coping strategies, *kinesiophobia* or the perception of illness, were not included. Additionally, the research included a large number of instruments, which might have fatigued the patients and reduced their motivation and focus when completing the questionnaire, and there is always a potential recall bias in patient-reported measures.

The findings of this research can contribute to a better understanding of the predictive factors of HRQoL in patients with CLBP and could be applied in clinical practice to improve CLBP management, focusing on psychological interventions. The results support and highlight the important role of psychological factors, which predict the physical and psychological dimensions of HRQoL. Moreover, the new results of this study can contribute to raising awareness among healthcare professionals about the important impact of psychological factors on all dimensions of HRQoL in patients with CLBP. In conclusion, these findings can play an important role in the creation of interventions in the treatment of CLBP based on the biopsychosocial model and focused on the person as a whole, not only on their physical health. A future research direction may be to create a longitudinal study or research based on psychological interventions related to factors that have been shown to be the predictors of HRQoL in patients with CLBP.

## 5. Conclusions

The results of the study identified a statistically significant correlation between the psychological and physical dimensions of HRQoL and all the investigated psychological factors: pain catastrophizing, pain-related anxiety, chronic pain acceptance, depression and anxiety. Furthermore, age, pain intensity, depression, pain-related anxiety and chronic pain acceptance stood out as significant predictors of the physical dimension of HRQoL, while pain intensity, anxiety and depression proved to be significant predictors of the psychological dimension of HRQoL in patients with CLBP. The results highlight the important role of psychological factors that predict the physical and psychological dimensions of HRQoL, and they can be called to action for incorporating psychological assessments and interventions in CLBP treatment.

## Figures and Tables

**Table 1:** Patients’ sociodemographic characteristics.

Characteristics	Value
Gender, *n* (%)	
Male	43 (21.4)
Female	158 (78.6)
Age (years), mean +/− SD	54.36 +/− 12.072
Residence, *n* (%)	
Urban	132 (65.7)
Rural	69 (34.3)
Working status, *n* (%)	
Employed	127 (63.1)
Unemployed	23 (11.4)
Retired	51 (25.5)
Marital status, *n* (%)	
Married	137 (68.1)
Divorced	20 (10.0)
Single	29 (14.4)
Widowed	15 (7.5)
Education level, *n* (%)	
Primary education	15 (7.5)
Secondary education	136 (67.7)
Tertiary education	50 (24.8)
Pain duration	
<1 year	38 (18.9)
1–3 years	32 (15.9)
4–6 years	28 (13.9)
>7 years	103 (51.3)

**Table 2:** Distribution of questionnaire scores.

Variables	Mean +/− SD	Minimum	Maximum
NRS	6.5 +/− 1.722	2	10
SF-PhyH	32.27 +/− 14.590	3.75	78.13
SF-PsyH	44.08 +/− 20.433	0	93.25
PSC	25.06 +/− 11.201	1	52
PASS-20	48.94 +/− 20.822	4	100
CPAQ-8	34.95 +/− 6.347	18	48
HADS-A	8.49 +/− 4.371	0	19
HADS-D	7.36 +/− 3.954	0	18

SF-PhyH—Health Status Questionnaire-Physical Health, SF-PsyH—Health Status Questionnaire-Psychological Health, NRS—Numeric Pain Rating Scale, PSC—Pain Catastrophizing Scale, PASS-20—Pain Anxiety Symptoms Scale Short Form, CPAQ-8—Chronic Pain Acceptance Questionnaire, HADS-A—Hospital Anxiety and Depression Scale (HADS)-Anxiety, HADS-D—Hospital Anxiety and Depression Scale (HADS)-Depression.

**Table 3:** Pearson’s correlation coefficients among the measured variables.

	SF-PhyH	SF-PsyH	Age	NRS	PSC	PASS-20	CPAQ-8	HADS-A	HADS-D
SF-PhyH	1								
SF-PsyH	0.74 *	1							
Age	−0.20 *	−0.11	1						
NRS	−0.53 *	−0.42 *	0.06	1					
PSC	−0.52 *	−0.51 *	0.04	0.38 *	1				
PASS-20	−0.56 *	−0.50 *	−0.01	0.44 *	0.68 *	1			
CPAQ-8	0.32 *	0.31 *	−0.07	−0.08	−0.12	−0.19 *	1		
HADS-A	−0.59 *	−0.70 *	0.12	0.27 *	0.62 *	0.626 *	−0.29 *	1	
HADS-D	−0.61 *	−0.65 *	0.17 *	0.29 *	0.51 *	0.509 *	−0.41 *	0.76 *	1

SF-PhyH—Health Status Questionnaire-Physical Health, SF-PsyH—Health Status Questionnaire-Psychological Health, NRS—Numeric Pain Rating Scale, PSC—Pain Catastrophizing Scale, PASS-20—Pain Anxiety Symptoms Scale Short Form, CPAQ-8—Chronic Pain Acceptance Questionnaire, HADS-A—Hospital Anxiety and Depression Scale (HADS)-Anxiety, HADS-D—Hospital Anxiety and Depression Scale (HADS)-Depression, *—*p* < 0.05.

**Table 4:** Summary of linear regression analysis for the dependent variable SF-PhyH.

R^2^		Adjusted R^2^		
0.576		0.561		
Predictors	B	β	t	*p*-Value
(Constant)	66.042		10.941	<0.0001 *
Age	−0.130	−0.107	−2.247	0.024 *
NRS	−2.725	−0.321	−6.196	<0.0001 *
HADS-A	−0.450	−0.135	−1.656	0.099
HADS-D	−0.915	−0.248	−3.274	0.001 *
PASS-20	−0.104	−0.148	−2.119	0.035 *
CPAQ-8	0.250	−0.109	2.146	0.033 *
PSC	−0.084	−0.064	−0.947	0.345

SF-PhyH—Health Status Questionnaire-Physical Health, NRS—Numeric Pain Rating Scale, HADS-A—Hospital Anxiety and Depression Scale (HADS)-Anxiety, HADS-D—Hospital Anxiety and Depression Scale (HADS)-Depression, PASS-20—Pain Anxiety Symptoms Scale Short Form, CPAQ-8—Chronic Pain Acceptance Questionnaire, PSC—Pain Catastrophizing Scale, *—*p* < 0.05.

**Table 5:** Summary of linear regression analysis for the dependent variable SF-PsyH.

R^2^		Adjusted R^2^		
0.577		0.563		
Predictors	B	β	t	*p*-Value
(Constant)	76.964		9.122	<0.0001 *
Age	0.002	0.001	0.030	0.976
NRS	−2.638	−0.222	−4.294	<0.0001 *
HADS-A	−2.134	−0.458	−5.616	<0.0001 *
HADS-D	−0.981	−0.190	−2.509	0.013 *
PASS-20	−0.039	0.040	0.573	0.567
CPAQ-8	0.290	0.090	1.782	0.076
PSC	−0.110	−0.061	−0.890	0.345

SF-PhyH—Health Status Questionnaire-Physical Health, NRS—Numeric Pain Rating Scale, HADS-A—Hospital Anxiety and Depression Scale (HADS)-Anxiety, HADS-D—Hospital Anxiety and Depression Scale (HADS)-Depression, PASS-20—Pain Anxiety Symptoms Scale Short Form, CPAQ-8—Chronic Pain Acceptance Questionnaire, PSC—Pain Catastrophizing Scale, *—*p* < 0.05.

## Data Availability

The data presented in this study are available on request.
